# A Real-Time PPG Peak Detection Method for Accurate Determination of Heart Rate during Sinus Rhythm and Cardiac Arrhythmia

**DOI:** 10.3390/bios12020082

**Published:** 2022-01-29

**Authors:** Dong Han, Syed Khairul Bashar, Jesús Lázaro, Fahimeh Mohagheghian, Andrew Peitzsch, Nishat Nishita, Eric Ding, Emily L. Dickson, Danielle DiMezza, Jessica Scott, Cody Whitcomb, Timothy P. Fitzgibbons, David D. McManus, Ki H. Chon

**Affiliations:** 1 Department of Biomedical Engineering, University of Connecticut, Storrs, CT 06269, USA; dong.han@uconn.edu (D.H.); fahimeh.mohagheghian@uconn.edu (F.M.); andrew.peitzsch@uconn.edu (A.P.); 2 Department of Biomedical Engineering, Johns Hopkins University, Baltimore, MD 21218, USA; sbashar3@jhu.edu; 3 BSICoS Group, Aragon Institute of Engineering Research (I3A), IIS Aragon, University of Zaragoza, 50018 Zaragoza, Spain; jlazarop@unizar.es; 4 Biomedical Research Networking Center in Bioengineering, Biomaterials and Nanomedicine (CIBER-BBN), 28029 Madrid, Spain; 5 Department of Public Health Sciences, University of Connecticut Health, Farmington, CT 06030, USA; nishita@uchc.edu; 6 Division of Cardiology, University of Massachusetts Medical School, Worcester, MA 01655, USA; eric.ding@umassmed.edu (E.D.); danielle.dimezza@aol.com (D.D.); jessicascott723@gmail.com (J.S.); timothy.fitzgibbons@umassmemorial.org (T.P.F.); david.mcmanus@umassmed.edu (D.D.M.); 7 College of Osteopathic Medicine, Des Moines University, Des Moines, IA 50312, USA; emily.l.dickson@dmu.edu; 8 School of Medicine, Tufts University, Medford, MA 02155, USA; cody.whitcomb@tufts.edu

**Keywords:** adaptive thresholding, atrial fibrillation, atrial fibrillation with rapid ventricular response, peak detection, peak correction, photoplethysmograph, premature atrial contraction, premature ventricle contraction

## Abstract

Objective: We have developed a peak detection algorithm for accurate determination of heart rate, using photoplethysmographic (PPG) signals from a smartwatch, even in the presence of various cardiac rhythms, including normal sinus rhythm (NSR), premature atrial contraction (PAC), premature ventricle contraction (PVC), and atrial fibrillation (AF). Given the clinical need for accurate heart rate estimation in patients with AF, we developed a novel approach that reduces heart rate estimation errors when compared to peak detection algorithms designed for NSR. Methods: Our peak detection method is composed of a sequential series of algorithms that are combined to discriminate the various arrhythmias described above. Moreover, a novel Poincaré plot scheme is used to discriminate between basal heart rate AF and rapid ventricular response (RVR) AF, and to differentiate PAC/PVC from NSR and AF. Training of the algorithm was performed only with Samsung Simband smartwatch data, whereas independent testing data which had more samples than did the training data were obtained from Samsung’s Gear S3 and Galaxy Watch 3. Results: The new PPG peak detection algorithm provides significantly lower average heart rate and interbeat interval beat-to-beat estimation errors—30% and 66% lower—and mean heart rate and mean interbeat interval estimation errors—60% and 77% lower—when compared to the best of the seven other traditional peak detection algorithms that are known to be accurate for NSR. Our new PPG peak detection algorithm was the overall best performers for other arrhythmias. Conclusion: The proposed method for PPG peak detection automatically detects and discriminates between various arrhythmias among different waveforms of PPG data, delivers significantly lower heart rate estimation errors for participants with AF, and reduces the number of false negative peaks. Significance: By enabling accurate determination of heart rate despite the presence of AF with rapid ventricular response or PAC/PVCs, we enable clinicians to make more accurate recommendations for heart rate control from PPG data.

## 1. Introduction

Algorithms for peak detection in photoplethysmogram (PPG) signals have been in use for a long time. PPG sensors have been primarily used in controlled clinical environments to obtain heart rates (HR) and oxygen saturation information from pulse oximeter devices. The peak detection algorithms embedded in commercially-available pulse oximeters are mostly designed for normal sinus rhythm (NSR) [1].

Smartwatches, which contain back-mounted light emitting diodes, enable near-continuous PPG data collection and potential novel mobile approaches to heart rhythm monitoring. Recently, there has been considerable interest in using smartwatches for detecting and monitoring patients with arrhythmias, including the world’s most common arrhythmia, atrial fibrillation (AF), which can be intermittent, asymptomatic, and cause irregular heart rates [2,3]. Atrial fibrillation can be associated with a basal (normal) or high resting pulse, depending on the characteristics of the atrioventricular conduction system. Previously, peak detection algorithms for HR estimation using PPG signals from wearable devices were designed using data from healthy subjects in normal rhythm. Nonetheless, peak detection algorithms designed using smartwatch and smartphone data from normal subjects show reasonable accuracy (i.e., >95% sensitivity and PPV for detecting pulse in NSR [4]) in their HR results, even in the presence of AF, so long as the heart rate is low, since the randomness in the variability of heart rates is captured, even if PPG peak detections are not optimized [2,3]. However, what we have developed in our current work, which no other authors have reported, is the ability to capture accurate PPG peak detection of fast heart rates (that is, to accurately estimate heart rates) due to either rapid ventricular responses, or premature atrial or ventricular contractions, using smartwatches. For these fast heart rates, the existing PPG peak detection algorithms will fail to provide accurate heart rate estimation, as they are not designed for this additional characteristic of AF or these other rhythms. In summary, existing PPG-based rhythm analysis algorithms may be adequate for AF detection but may not provide accurate heart rate estimation for patients in AF with fast heart rates due to rapid ventricular responses, or patients with premature atrial/ventricular contractions. This is an important limitation since resting heart rate control is a key treatment target for AF patients.

There exist many excellent peak detection algorithms for normal sinus rhythm [4,5,6,7]. The use of empirical mode decomposition (EMD) for peak detection of NSR has been effective, usually in the event of significant respiratory effect on the PPG signal [8]; however, it cannot be used to automatically detect accurate heart rates during an episode of AF interrupting NSR, for example. Peak detection algorithms based on the morphology of the PPG waveforms are accurate provided that motion and noise artifacts are minimal, as they can affect the morphology of the signal [5]. Moreover, because of these algorithms’ reliance on the morphology of the PPG waveforms, the algorithms have had application primarily to NSR [5]. A different method by Shin et al. [4] uses an adaptive threshold method to detect maxima of PPG pulses, however, the algorithm’s accuracy can degrade if both the incident and reflected waves of the PPG merge, which can occur with disease conditions such as AF. Recently, Harju et al. [7] and Renevey et al. [9] proposed an algorithm for heart rate monitoring of AF and NSR subjects, however, it is not designed for fast heart beats that are interspersed with normal rates. The amplitude and velocity of the reflected PPG wave will rapidly change depending on the randomness of AF rhythms, especially when basal heart rates are high (e.g., >140 beats/min). This is one of the main problems any peak detection algorithm has to overcome, particularly for complicated rhythms such as AF. Compounding this problem is the poor signal-to-noise ratio (SNR) of the PPG signal in some subjects, which can be caused by either their darker skin or poor perfusion of arterial blood. Certainly, motion artifacts will cause a poor SNR, hence, any algorithm that is able to discriminate among the above-listed heart arrhythmias must start with a relatively clean signal. However, automatic detection of motion artifacts that relies on accelerometers will be ineffective when a smartwatch is not worn tightly (causing some gap between LEDs and the skin), even if the wrist is not moving.

For all these reasons, it is difficult to design an algorithm that can automatically account for the normal heartbeats of NSR, the fast heartbeats associated with premature ventricular contraction (PVC) or premature atrial contraction (PAC), and the rapid ventricular response (RVR) or basal (normal) heart rates of AF patients. No doubt owing to these challenging scenarios in PPG signals, we are not aware of any published PPG peak detection algorithms that can automatically provide an accurate heart rate for subjects with NSR, PAC, PVC, or AF with both normal and fast heart rates.

Clinically, it is not only important to detect AF, but accurate heart rate estimation in AF is critical, since high rates are associated with adverse long-term outcomes and symptoms. Furthermore, atrioventricular nodal blocker agents are commonly prescribed for AF patients to reduce or control the ventricular response to AF [10]. Many patients with AF are prescribed conventional ECG monitors to assess the quality of heart rate control and we envision a use-case scenario where PPG-based systems may be used for the same purpose. Therefore, it is important not only to discriminate AF from NSR, but also from other rhythms such as PAC or PVC from PPG [11,12,13], and to determine an accurate heart rate among individuals with AF.

In this paper, our main contributions are:

1. Our proposed algorithms were tested on three different models of smartwatches: Samsung’s Simband, Gear S3, and Galaxy Watch 3. We trained our algorithms only on the PPG data recorded from Simbands, and independently tested them with large PPG datasets consisting of PPG signals from Gear S3 and Galaxy Watch 3 smartwatches. This is the main difference from our previous published conference proceedings in [14].
2. Our proposed algorithms showed reliable results not only for NSR data, but also for various cardiac arrhythmias such as PAC/PVC, basal AF, and AF with RVR.
3. Unlike deep learning algorithms that only track heart rates on NSR subjects [15,16,17], our algorithm is computationally efficient and was embedded in Samsung Gear S3 and Galaxy Watch 3 smartwatches for heart rate estimation and AF detection in an NIH-funded clinical trial (study ID NCT03761394).

To the best of our knowledge, this is one of the first studies [14] demonstrating that a real-time PPG peak detection algorithm can provide accurate estimation of heart rates despite various challenging cardiac arrhythmias including not only NSR but also AF and PAC/PVC. In addition, the algorithm is shown to be robust on different generations of models of smartwatches from Samsung Corporation.

## 2. Materials and Methods

### 2.1. Dataset and Experiment Protocols

#### 2.1.1. Peak Detection Strategy and Motivation

**Figure 1:**
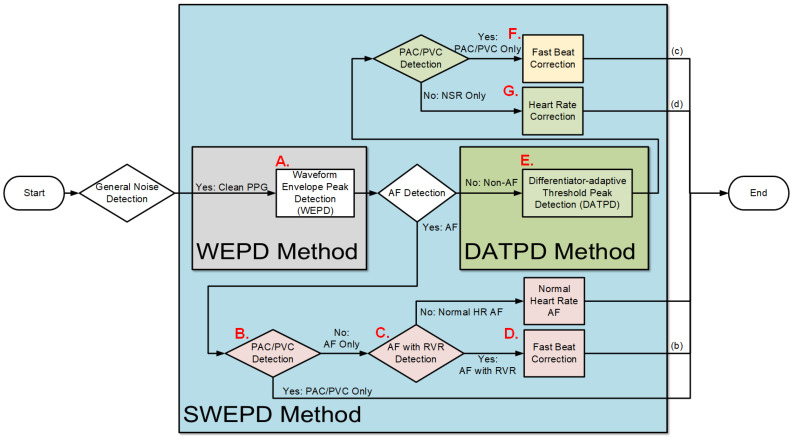
Flow chart of the proposed sequence including our new peak detection algorithm and a new peak correction method. Step A: perform PPG peak detection on the clean PPG using the WEPD peak detection algorithm described in Section 2.2.2. Step B: if AF detected, perform PAC/PVC detection on the derivatives of heart rates to ensure AF was not falsely detected, as described in Section 2.2.3. Step C: perform AF with RVR detection on the Step B segments without PAC/PVC, as described in Section 2.2.4-1. Step D: perform fast beat correction on Step C segments with RVR, as described in Section 2.2.4. Step E: perform peak detection algorithm on the clean PPG using DATPD algorithm described in Section 2.2.5. Step F: perform fast beat correction on segments with only PAC/PVC, as described in Section 2.2.6-1. Step G: perform heart rate correction on the NSR segments, as described in Section 2.2.6-2. Steps (b)–(d) represent the steps of additional heart rate correction on the output of WEPD peak detection.

To be able to discriminate among the various rhythms, we developed a sequence of peak detection algorithms, each specifically designed for a particular arrhythmia (or lack thereof), shown in Figure 1. The main assumption of the method is that we are using a clean PPG data segment, for the reasons described above. Determination of clean PPG is provided by the motion artifact detection algorithm described in [18]. Since PPG waveforms from AF are vastly different from those of NSR, the sequence must start by distinguishing between these two rhythms. As AF detection in a PPG signal must start with a peak detection algorithm, the accuracy of which is not as important as capturing the randomness of the heart rate variability, in theory any of the previously developed peak detection algorithms [5] could be used for the first stage of the sequence. However, we developed an improved PPG peak detection algorithm, which we describe in detail in the Methods section and compare its performance to one of the more accurate previous algorithms [5]. The new peak detection algorithm is termed waveform envelope peak detection (WEPD) for PPG signals. WEPD is specifically designed to reduce false negative and false positive detection of AF and NSR, respectively, which consequently results in better performance when compared to a typical previously developed peak detection algorithm, as shown in the Methods and Results sections. The peak algorithm which we compared to is based on the morphology of PPG waveforms [5]. As it was lacking a name, we termed it the differentiator-adaptive threshold peak detection (DATPD) algorithm [5]. SWEPD is the overall algorithm which combines both DATPD and WEPD so that various arrhythmias can be accounted for, to determine heart rates more accurately. Thus, depending on the type of arrhythmia detected, the PPG peak detection may be comprised of the following combinations: only WEPD is included in SWEPD when PPG data are detected as AF after general noise detection; or WEPD and DATPD are both included in SWEPD when PPG data are detected as non-AF after general noise detection.

For both AF and other rhythms, the next step is to correct for PAC/PVC patterns, if they exist. For detecting PAC/PVC episodes, we use a Poincaré plot of the derivative of heart rates, which delineates patterns that are unique to these rhythms. The next step involves discriminating between fast and basal heart rate cases to perform an accurate peak location determination. These comprehensive sequential steps are used to discriminate between NSR, PAC/PVC, and RVR and basal heart rate AF rhythms. The overall sequence using WEPD, henceforth, is named SWEPD. The evaluation of our proposed SWEPD method was based on data collected from both NSR and AF subjects using Samsung’s Simband 2 smartwatches at the University of Massachusetts Medical Center (UMMC).

#### 2.1.2. Training Dataset: UMMC Simband Dataset

35 participants (28 male and 7 female) with cardiac arrhythmia ranging in age from 50 to 91 years old participated in the smartwatch study at the ambulatory cardiovascular clinic at University of Massachusetts Medical Center (UMMC). The Institutional Review Boards at both UMMC and the University of Connecticut approved the study protocol. Reference ECG and smartwatch data were measured simultaneously from the chest and a wrist using a 7-lead Holter monitor (Rozinn RZ153+ Series, Rozinn Electronics Inc., Glendale, NY, USA) and a smartwatch (Simband 2, Samsung Digital Health, San Jose, CA, USA (henceforth referred to simply as Simband)), respectively. ECG data consist of 3-channel signals, each sampled at 180 Hz [19]. The Holter ECG data were used as the reference for estimation of heart rates. Simband data consist of 8-channel PPG signals and three axis accelerometer signals. Note that while a one-lead ECG can be obtained by touching the Simband wristband’s stainless-steel electrode, we only used that data for signal alignment between Simband and Holter data at the start of the experiment. Simband PPG signals were sampled at 128 Hz. Only the 5th PPG channel (green LED color, wave length 520–535 nm) was used for data analysis since it consistently provided the best signal quality, and this was recommended by Samsung [20]. The subjects were asked to touch the Simband’s ECG electrode for at least one minute at the beginning of data collection so that PPG data could be aligned with Holter ECG data.

The alignment of the Simband and Holter ECG signals was performed by estimating the cross-correlation between the signals [21] When two signals are well-aligned, the cross-correlation value will have a value >0.8 and if they are not, it can be lower than 0.4. However, even for perfectly aligned signal, the cross-correlation value will be lower than 1 if they have a low signal-to-noise ratio. For this reason, we chose 0.9 for the alignment of ECG data from Simband and Holter monitors. We manually checked both ECG signal at the point where alignment could occur when cross-correlation is around 0.9, and we slightly adjusted the alignment time point to make sure R waves in both ECG signals were precisely aligned. Given this need for correlation, only the Holter ECG data with good signal quality (discernible R waves) were retained, as they also needed to be used as the reference heart rates from which PPG heart rate estimates were benchmarked. PPG and accelerometer data were down sampled to 50 Hz and 30 Hz, respectively. All signals were segmented into 30-s lengths, with no overlap, for peak detection analysis in real time clinical detection. This dataset was created as a part of a preliminary study, thus we have a limited number of subjects and recordings. The training dataset is available for download on our lab’s website listed in https://biosignal.uconn.edu/resources/ (accessed on 30 June 2021).

#### 2.1.3. Experiment Protocol for the Training Dataset

The study protocol was designed to simulate activities of daily living during smartwatch use and consisted of the following sequence: sit still for 2 min, walk slowly for 2 min, stand still for 30 s, walk quickly for 2 min, stand still for 1 min, move arms randomly for 2 min, and stand still for 30 s. Participants were then asked to sit and stand repetitively for 1 min followed by climbing stairs for 2 additional minutes. The last procedure required participants to sit for 1 min. The estimated time to finish the entire protocol is 14 min, and 50% of the protocol consists of physical movements. All participants signed an informed consent, and study procedures underwent review and were approved by the University of Massachusetts Medical School (UMMS) Institutional Review Board (IRB ID: H00009953).

#### 2.1.4. Testing Dataset: UMMC Pulsewatch Dataset (Samsung Gear S3 and Galaxy Watch 3)

For the independent testing dataset, we randomly selected 25 subjects from participants enrolled in the ongoing clinical trial (funded by NIH, study ID NCT03761394) at UMMC. The subjects are from the same age group (age > 50 years old) as the training dataset except for a history of stroke or transient ischemic attack (TIA). All participants were asked to simultaneously wear a 1-lead reference ECG patch device (Cardea SOLO, Cardiac Insight Inc., Bellevue, WA, USA) and a smartwatch. The smartwatch used at the start of the clinical trial was Samsung Gear S3 (Samsung, San Jose, CA, USA) (henceforth referred to simply as Gear S3). During the second half of the clinical trial, the smartwatch was gradually switched to Samsung Galaxy Watch 3 (Samsung, San Jose, CA, USA) (simply referred to as Galaxy Watch 3) due to the issue of battery health degradation of Gear S3 and the discontinuity of Gear S3 on the market. Both Gear S3 and Galaxy Watch 3 are different than the Simband we used for the training data collection. The patch ECG data consisted of 1-channel signals and were sampled at 250 Hz and were used as the reference. The Gear S3/Galaxy Watch 3 data consisted of 1-channel PPG signal and 1-channel magnitude of the accelerometer signal. Gear S3/Galaxy Watch 3 signals were all sampled at 50 Hz and were automatically segmented into 30-s lengths. The alignment of the Gear S3/Galaxy Watch 3 and ECG patch signals for every 30-s segment was done by cross-correlation and visual inspection. The reference ECG heart rates calculated from the patch ECG was based on Hossain et al.’s method [22].

The enrolled patients wore the Gear S3/Galaxy Watch 3 smartwatch and ECG patch 24 h a day with no restriction on their regular daily activities, for 14 consecutive days, to evaluate the performance of our real-time PAC/PVC detection algorithm. Due to the 7-day battery limitation, patients switched to a second new ECG patch on the 7th day of trial. Smartwatches were charged daily for 1 h, twice a day.

#### 2.1.5. Experiment Protocol for the Testing Dataset

All testing data were collected from “Pulsewatch” smartwatches worn by the participants in the intervention group in Part II of our clinical trial study (NIH study ID: NCT03761394), which was a 14-day at-home, free-living protocol [23]. The participants wore a continuous AF monitoring ECG patch, approved by the FDA, which did not restrain their daily activities [23] and allowed us to collect a reference ECG signal. The “Pulsewatch” system was developed in Part I of our clinical trial study, and consisted of a smartwatch (Samsung Gear S3 or Galaxy Watch 3) and a smartphone (Samsung Galaxy S6, S10e, A50, or XCover Pro) pre-installed with our own applications for data collection and analysis. Only the smartwatch application software performed all signal processing tasks in near real-time including motion artifact and AF detection and our proposed PPG peak detection and PAC/PVC detection [18]; the smartphone application functioned as a data transfer hub to upload PPG data to our secure cloud server. Written informed consent forms were collected from all patient participants, and formal ethical approval for this study was obtained from the UMMS Institutional Review Board (IRB ID: H00016067) [23].

### 2.2. Methods and Evaluation

#### 2.2.1. Overview of the Proposed (SWEPD) Method

Figure 1 shows the diagram of our proposed new peak detection method. Motion artifact detection is performed as the first step to ensure that PPG data segments are relatively clean (≤3 s of motion artifacts). Details of the motion artifact detection algorithm used have been described in [18]. To succintly summarize the criteria of detecting a watch segment as noisy, ref. [18] used both accelerometer (ACC) and PPG signals to decide if motion and noise artifacts existed more than 3-s in a 30-s PPG segment. The algorithm involves a combination of features derived from a time-frequency analysis and the significance of the accelerometer data’s amplitude. How we derived all threshold values is provided in [18]. In SWEPD, as motion artifacts in PPG signals can lead to incorrect peak detection, threshold values are chosen that classify even moderately corrupted data (>3-s of motion artifacts) as unusable data.

If the PPG data pass the motion artifact detection test, peak detections are performed first by using the waveform envelope peak detection (WEPD) algorithm. The WEPD algorithm is similar to standard PPG detection algorithms, which have been designed largely for normal sinus rhythm, but the method differs in that it is designed to ignore false positive beats caused by the dicrotic notch while retaining sensitivity to irregular heartbeats in AF data.

The next step involves AF detection [18] to separate AF from non-AF segments. For AF-detected data segments, the peak detection results from the WEPD algorithm are retained. This is because the WEPD algorithm is designed to capture irregular beats that are the hallmark of AF. The next steps are to apply a PAC/PVC detection algorithm followed by classification into RVR or basal heart rate AF. For non-AF detected segments, we use the differentiator-adaptive threshold peak detection (DATPD) algorithm [5,24] which is primarily designed to detect normal sinus rhythm. Consequently, DATPD algorithm, due to its adaptive thresholding is not designed to detect PAC/PVC beats. Subsequent steps involve detection of PAC/PVC patterns and if detected, a beat correction algorithm is applied to account for the fast heart rates [25]. Details of each of these steps are provided in the next sections.

#### 2.2.2. Details of New WEPD Peak Detection Algorithm

**Figure 2:**
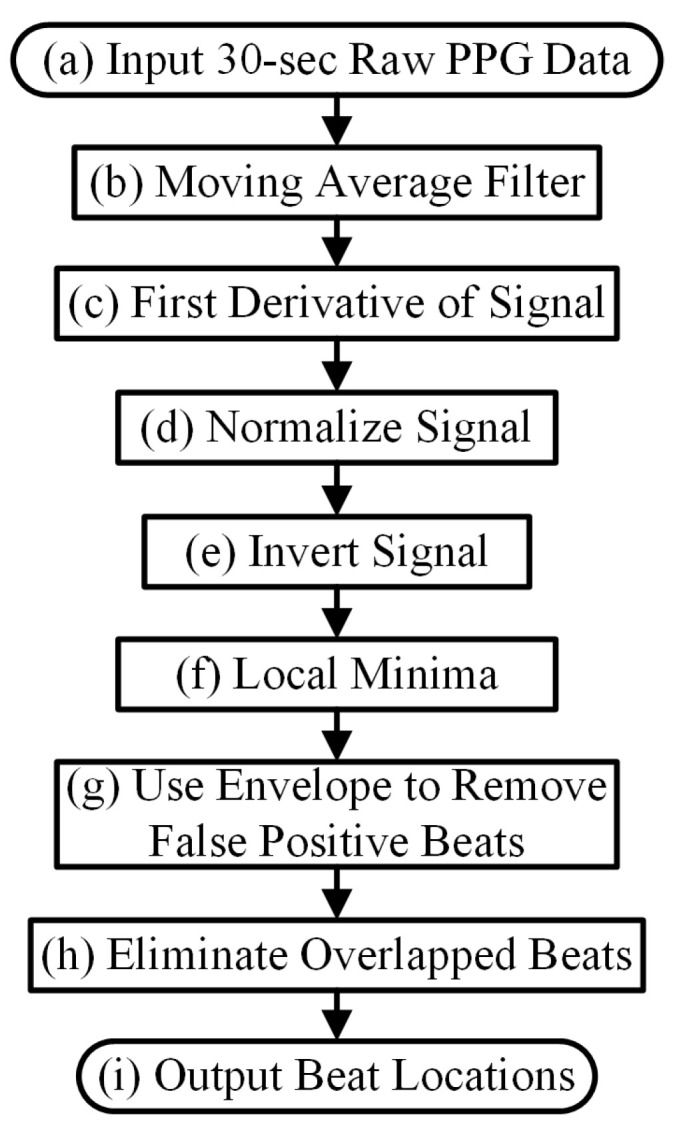
Flowchart of the WEPD algorithm.

This is step A in Figure 1. The overall flowchart of the WEPD algorithm is provided in Figure 2. We describe each of the steps in the next sections.

1. Moving Average Denoising and Differentiation
  A bandpass filter with a passband of 0.5 Hz to 5 Hz is applied to each 30-s PPG data segment that has been declared to be relatively devoid of motion artifacts. We used a 5th order zero phase elliptic filter to design our bandpass filter. The design of this filter is based on the PPG pre-processing section mentioned in Chong et al. [6], which was optimized on smartphone PPG signals. The bandpass filter does not ensure smoothness of the signal, thus, a moving average filter with a proper sliding window is used to remove non-cardiac related oscillations and spikes which may have been caused by motion artifacts or poor signal quality data. This smoothing process is implemented three separate times:
  (1) $$b\left(i-M+1\right)=\frac{1}{2M+1}\sum_{j=i-M}^{i+M}a\left(j\right)$$
  where i=M,M+1,M+2,…,N−M−1 with M=fs/10 (round *M* to nearest integer if *M* is not an integer) for the first moving average process, and M=fs/9 (round *M* to nearest integer if *M* is not an integer) for the remaining two times; denotes the raw signal after bandpass filtering; and fs is the sampling rate.
  Before the final smoothing, calculation of the first-order difference of the filtered signal is performed to accentuate small fluctuations in the peak plateau portion of the PPG waveform:
  (2) c(i)=b(i+1)−b(i)
2. Standardizing PPG
  We subtract the mean of the signal from the above step, and then divide it by its standard deviation.
  (3) $$d\left(i\right)=\frac{c(i)-\mathrm{mean}(c)}{\mathrm{std}(c)}$$
  This step is especially important to enable the signal’s waveform envelope calculation to remove false positive beats, which will be detailed in the subsequent section.
3. Inverting the Signal and Performing Peak Detection
  Peak detection is applied on either the PPG pulses’ top or bottom curve portion that has the sharpest peaks, to maximize the accuracy of heart rate estimation. To determine which side has the sharpest peaks (either top or bottom portion of a PPG segment), we first eliminate the side with detected number of peaks that are more than the fastest heart rate a human could have. If both top and bottom portions are relatively clean, we use the peaks from the side (top or bottom) with fewer beats. If both top and bottom portions have similar number of beats, we choose the side of the PPG has the sharper peaks by using finding the side has larger mean value of amplitude gradient around the peaks. If the top portion of the PPG signal is chosen, we flip the signal and estimate the local minima points for each beat. Example of inverting of PPG is in the panel (d) and (e) of Figure 3.

**Figure 3:**
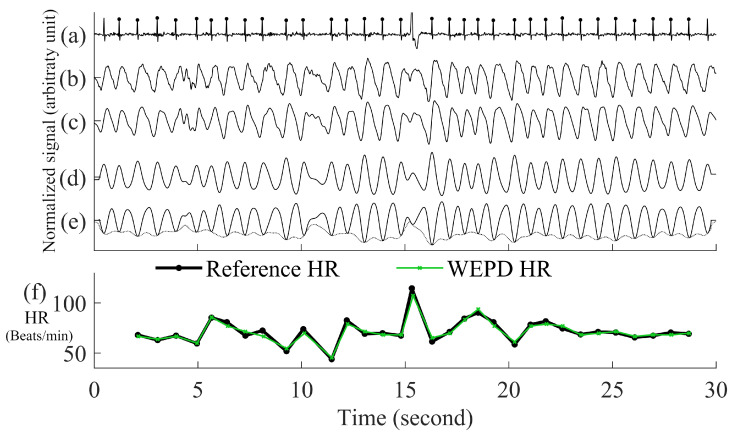
Step by step procedures for the proposed WEPD algorithm on a basal heart rate AF signal. The top panel (**a**) is the raw ECG signal with the reference ECG beats; panel (**b**) is a raw PPG signal, panel (**c**) is the output of the bandpass filter; panel (**d**) is the output after moving average filtering and the first derivative operation; panel (**e**) is the output after signal standardization, inversion of the signal, and application of the envelope method for local minima detection. The bottom panel (**f**) is a comparison of the heart rate (unit: beats per minute, BPM) from the reference ECG signal vs. estimated heart rate from WEPD.
4. Removing False Positive Peaks Using an Envelope
  For high resolution PPG data from a commercial grade pulse oximeter and Simband, a dicrotic notch is prevalent in most subjects with normal sinus rhythm. Motion artifacts in the wrist can also create the appearance of a dicrotic notch. If a true or artifact dicrotic notch is not accounted for, our peak detection algorithm will detect it as a heartbeat. Consequently, this will lead to erroneously fast heart rate detection and depending on the rate of appearance of dicrotic notches, it can also lead to false detection of AF. Hence, to disregard any dicrotic notches, we estimate the envelope of the bottom portion of each beat of the PPG signal. There are two ways to calculate the envelope of the PPG signal. The first way is to interpolate the local minimas in the PPG signal with a cubic spline. This method has been used for respiratory rate detection with PPG signals [26], and it can accurately capture the general outlines of the PPG waveform. The second way is to use an N-tap Hilbert filter to obtain the upper envelope of the analytic signal; this method has been successfully used for abnormal heart sound analysis [27]. For the length of “N-tap” in the Hilbert filter, we found that it works the best on a smartwatch PPG signal with the value of 1.5 times the sampling frequency of PPG. This defined N-tap filter allows the envelope to account for dominant grooves of PPG waveforms. For each beat, the minimal amplitude that intersects between the PPG signal and two envelopes is considered as the new estimated peak.
5. Eliminating Overlapping Peaks
  If we obtain more than one intersection point between each beat of the PPG signal and its envelope, we retain the lowest amplitude peak and disregard the other candidate peaks. To ensure that we are not choosing two consecutive heartbeats (after going through steps (a)–(e) in Figure 3) that are too closely spaced, we stipulate that the time difference between two adjacent minima peaks has to be greater than 0.3 s, which is considered the refractory period of cardiac muscle for normal sinus rhythm [28]. A representative example of the PPG signal that has gone through the WEPD algorithm is shown in Figure 3. Note the accuracy (squared error of the mean HR: 0.21 BPM and the root mean squared error (RMSE) of beat-to-beat HR: 2.79 BPM) in the estimation of heart rates via the WEPD algorithm when compared to the reference ECG.

#### 2.2.3. PAC/PVC Detection

Now we are in step B in Figure 1. We use a PAC/PVC detection method based on an algorithm we previously developed to use on data obtained from a smartphone’s video camera [29,30]. We had to modify the algorithm to be applicable to a smartwatch; for example, a smartphone’s video signals are much cleaner than the smartwatch PPG. However, the principal behind the PAC/PVC detection is unchanged (Chong et al.) [6]. Essentially, the approach is to plot on the Y-axis the difference in heart rates between the current and the previous beats. Then, we plot on the X-axis the difference in heart rates between the current and the next beats. The plot is also subdivided into nine regions [25], which are associated with different heart rates and their patterns of repeatability. When this Poincaré plot is computed, there are unique patterns that are only associated with PAC/PVC. Details of our PAC/PVC detection method are provided in [25].

#### 2.2.4. New Peak Correction Method for AF Patients

**Figure 4:**
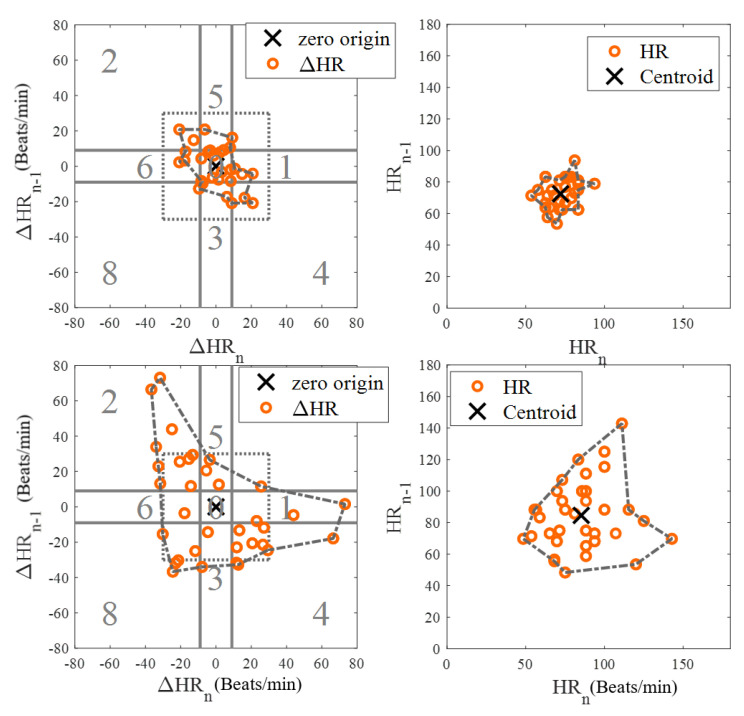
Poincaré plots for basal (**top panels**) and RVR (**bottom panels**) heart rate AF detection. The left panels are the Poincaré plot of heart rate difference, and the right panels are the Poincaré plot of heart rate. In the left panels, the entire Poincaré plot is separated into 9 regions, which are also used to locate erroneous peaks for peak correction during PAC/PVC detection.

1. AF with RVR Detection for AF Subjects
  Now we are in step C in Figure 1. The aim of this step is to detect AF with a rapid ventricular response, which will henceforth be called AF with RVR. Thus, we designed a supervised linear classifier to separate fast from normal heart rate AF. As this is one of the first studies to investigate AF with RVR in a smartwatch PPG, we define it based on the following criteria which all should be met:
  (a) A segment has to be an AF segment. In our algorithm, this segment has to be first detected as an AF segment from the AF detection algorithm [18] using the heart rates provided by the WEPD algorithm.
  (b) A heart rate that is greater than 140 BPM, based on our observation on the training data and [31,32,33]. For example, in [33] authors used 150 BPM as a threshold for defining faster heart rate among AF with RVR in the ICU; in [32] it was suggested that the upper limit is 130 BPM for target heart rate control among AF and heart failure patients during moderate exercise; in [31] author used 140 BPM as the minimum ventricular rate for marking patients with rapid ventricular rate in atrial fibrillation.
  (c) A heart rate that increases more than 40 BPM from one beat to the next. For example, ref. [34] suggested that the heart rate value cannot change more than 10 BPM from a previous value on normal sinus rhythm, and from our observation on our training data with PAC/PVC and AF with RVR subjects, we noticed that the heart rates for fast beats usually increased more than 20 BPM for the former, and more than 40 BPM for the latter.
  (d) The heart rate estimation from the WEPD is inaccurate for more than half the beats in each reference heart rate segment. WEPD is largely appropriate for determining irregularity of the rhythm, thus, accurate heart rate estimation requires SWEPD.
  Among 10 AF subjects with AF detected in 53 segments, 25 were labelled as RVR heart rate and 28 as basal heart rate AF, based on the criteria defined above. All 53 segments were used for determining a threshold value for fast heart rate detection. The top and bottom panels of Figure 4 show Poincaré plots of basal heart rate AF and AF with RVR, respectively. As shown in Figure 4, the basal AF heart rates are more confined to the origin whereas the AF with RVR heart rates are more dispersed. To exploit this main characteristic, we selected nine candidate features to characterize the AF with RVR segments. One feature is the number of beats exceeding 140 BPM, five other features are derived from the Poincaré plot of the heart rate difference (ΔHR_*n*_ and ΔHR_*n+1*_), and the remaining three features are derived from the Poincaré plot of heart rates (not difference of heart rates). The left panels of Figure 4 show Poincaré plots of the heart rate difference. The heart rate range for both X and Y axes are set to [−80, 80] BPM. Four of the five features derived from these plots are based on calculation of the distance of each point to the origin, from which we determine: the mean value of all distances, variance of all distances, boundary of the covered area of all points, and a ratio. This last is the ratio of points that have a distance to other points that is lower than 5 BPM to the number of combinations to calculate the distance between all of the points in the Poincaré plot. The fifth feature is the ratio of points that fall inside a confined square box (the dotted line square boxes in the left panels of Figure 4) to the entire number of points in the Poincaré plot; points outside of this dotted-line box indicate rapidly changing heart rates. To find the optimized limits of the confined box, we tested various heart rates ranging from [−10, 10] BPM to [−45, 45] BPM by increasing the X- and Y-axes by 5 BPM. The box’s confined limit of [−15, 15] BPM showed the best results to separate between the fast and normal heart rates of AF segments. The right panels of Figure 4 show the Poincaré plots without the difference of heart rates, which were derived from the WEPD approach. The X-axis represents the *n+1* heart rate and Y-axis represents the current heart rate. The heart rate boundary for both axes was set to [0, 180] BPM. The centroid of each point in the Poincaré plot is calculated by averaging coordinates of all points. The distance between the centroid and all points was then calculated. Three features were calculated from this distance: the mean of distance, variance of distances, and the area in the boundary of all points as shown in the right panel of Figure 4. Of the nine features described above, we found that the mean value of all distances from the Poincaré plot of heart rate difference was the best in separating the basal from the RVR heart rate AF. Figure 5 shows the threshold value to separate RVR and basal heart rate, which was found to be 22.4 BPM.

**Figure 5:**
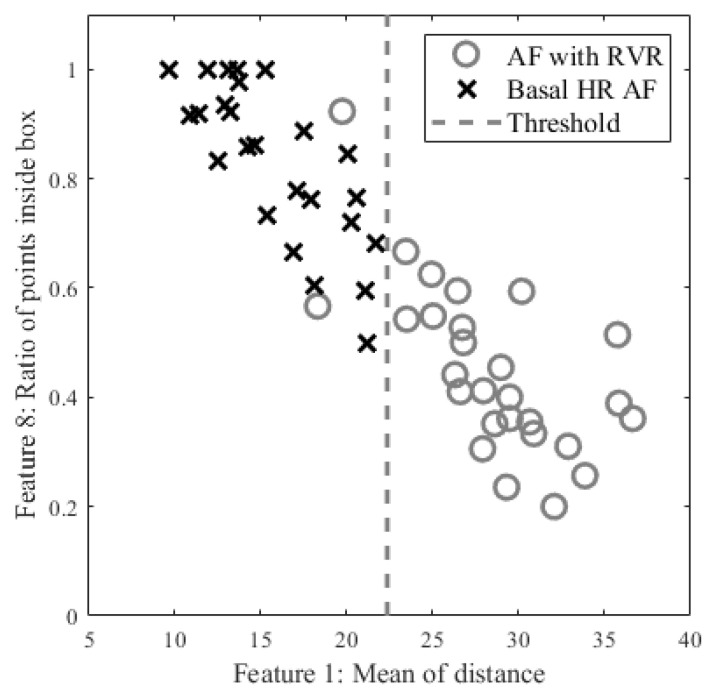
Scatter plot of candidate features for heart rate estimation among individuals with AF stratified by high vs. basal ventricular response. The X axis is the best feature value of mean of distance calculated from the Poincaré plot of heart rate difference; the Y axis is the second-best feature value of ratio of points inside a confined box from a Poincaré plot of heart rate.
2. Peak Correction for AF with RVR Segments
  In the last section, we described how the AF with RVR segments can be discriminated from the basal HR AF data. Given this information, the next task is to search for additional peaks in the PPG signal to address the AF with RVR data segments. In the left panels of Figure 4, we section the entire Poincaré plot into nine regions, which represent all possible permutations of basal and RVR heart rate changes within the three consecutive heartbeats [6]. We set the size of region zero to [−9, 9] BPM (this represents the limits of the normal heart rates), and the other region boundaries can be inferred from Figure 4. Region two represents a true scenario where a fast heartbeat is surrounded by two slow heartbeats. This is a typical scenario for a RVR heart rate AF. This section is to describe how we look for fast heart rates in step D in Figure 1 based on the progression of the flow chart. As detailed in the preceding section, when a heartbeat is located in the other 8 regions (other than the second region), it is incorrectly detected as a basal heartbeat. Thus, the next task is to re-examine a filtered PPG signal between two detected beats to look for additional local minima. The last row of Figure 6 illustrates the above-described concept. The heart rates estimated from the WEPD method are shown in gray circles. For each of these beats, we also label the regions they are associated with. The dark circle lines are the reference heart rates. As shown, the heartbeats labelled region two coincide with the reference heart rates, as expected. However, heartbeats labelled with other regions (1,3,4,5,7,8) deviate significantly from the reference heartbeats, as expected. Hence, these beats are all candidates for having been incorrectly detected as normal heart rates that need to be corrected if they are actually fast heart rates.
  To account for heart rates mis-detected as normal, we modify the WEPD method’s procedures. The first step involves increasing the upper band of the bandpass filter from [0.5, 5] Hz to [0.5, 10] Hz. This procedure will lead to additional peaks in the PPG signal, if they exist. Additionally, we assume that the fastest heart rate of an AF subject should be less than 200 BPM (based on data collected from AF subjects in this study), and that the refractory period of cardiac muscle for AF should be faster than the normal refractory period of 0.3 s [28]. Given these assumptions, any additional peaks detected via the increased bandpass filter will not be considered as candidates if they are less than 0.3 s apart from the original peak detected via the first pass of the WEPD algorithm prior to AF detection, as noted in Figure 1. If there is a peak that is greater than 0.3 s apart, a local minima (assuming the signal is already inverted to look for the minima points) detection is applied. If there is no local minima point, the middle time point of the candidate interval is selected as a new fast heartbeat. If there are several local minima points that are greater than 0.3 s apart from the original peak, we use the first local minima location as the new fast heartbeat. The aforementioned additional fast beat locations are combined with the peaks detected from the first pass of the WEPD algorithm’s results. Rows (c–d) in Figure 6 show the additional peaks detected using the second pass of the WEPD method via the modified bandpass filter ([0.5, 10] Hz), and the resultant heart rates, respectively. As shown in row (e) of Figure 6, the modified heart rates labelled as the gray crossed line match the reference heart rates significantly better than the heart rates derived from the first pass of the WEPD algorithm, shown as the gray circled line.

#### 2.2.5. Details of New Peak Detection for PAC/PVC Detection

We now move to the non-AF detected portion of the flowchart, which is marked as green and yellow background colors shown in the panel of Figure 1.

**Figure 6:**
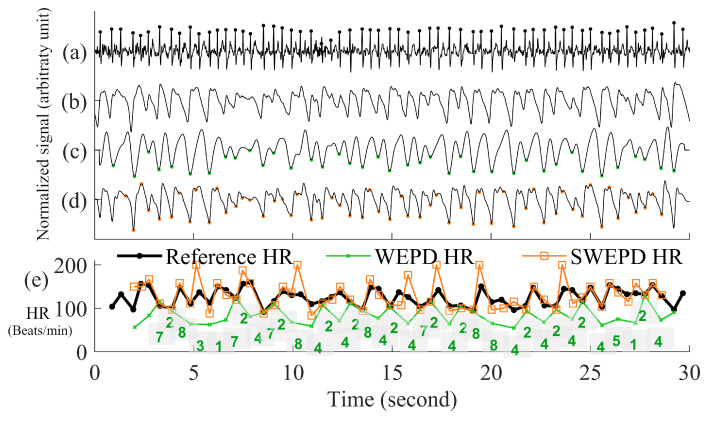
Representative example of peak correction for AF with RVR. Top panel (**a**) is the reference ECG; panel (**b**) is a raw PPG signal; panel (**c**) is the peak detection output from WEPD; panel (**d**) is the peak correction (SWEPD) for fast heart rate AF with corrected heartbeats. The bottom panel (**e**) is the heart rate (unit: BPM) comparison with the reference heart rates, estimated heart rates from SWEPD and peak corrections of fast heart rate AF. Region information for each heartbeat of WEPD is labeled on the heart rate in the bottom panel (**e**).

**Figure 7:**
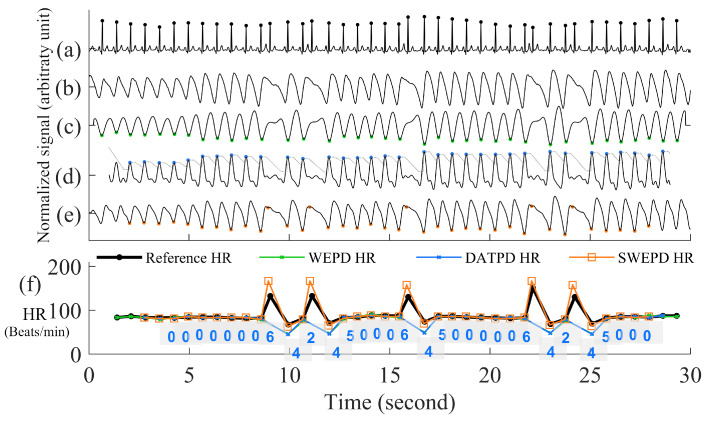
Representative example of the peak correction for fast heart rate PVC. Top panel (**a**) is reference ECG; panel (**b**) is the raw PPG signal; panel (**c**) is the peak detection output from WEPD; panel (**d**) is peak detection from DATPD; panel (**e**) is the peak correction for PAC/PVC with corrected heartbeats (unit: BPM) via SWEPD. The bottom panel (**f**) is heart rate comparison with the reference heart rate, estimated heart rates from WEPD, DATPD, and heart rates from the peak correction of PAC/PVC. Region information for each heartbeat of DATPD peak detection is labeled on the heart rate in bottom panel (**e**).

1. The DATPD Algorithm
  Now we are in the step E in Figure 1. This section describes an approach that better reflects heart rate patterns of NSR including PAC/PVC using a similar approach previously described [5]. The motivation for the approaches for better peak detection even with PAC/PVC or fast NSR beats is shown in Figure 7. The top row (panel (a)) shows an example of PVC beats. The panel (b) shows the peak detection estimated from the WEPD method, while the WEPD does well in detecting most of the PVCs, it incorrectly assigned two beats for the 4th PVC beat instead of a single beat. To mitigate this issue, even for PAC/PVC and the fast heart rate NSR beats, we describe in the next section various procedures to overcome low heart rate estimation in the event of fast heart rates.
2. Reconstruction of Clean Signal Using VFCDM
  Lázaro et al. used a FIR low-pass-differentiator filter with the pass band (0–7.8 Hz) [5] to filter the PPG signal. However, this linear filter was specifically designed for fingertip PPG signals. The motion artifact characteristics of a smartwatch PPG are often observed in the frequency band from 3.5 Hz to 7.8 Hz [35,36]. Moreover, the differentiator filter will accentuate the rapid changes in the PPG signal caused by motion artifacts, resulting in erroneous high amplitude peaks in the first derivative of the filtered signal. Consequently, an adaptive threshold method to detect peaks becomes more difficult. To overcome these issues, we illustrate our preferred approach.
  The first step involves the use of a time-frequency technique named Variable Frequency Complex Demodulation Method (VFCDM) [37]. The VFCDM technique has been described in detail and tested with different physiological signals [38]. Only the first two components are within a realistic heart rate range (e.g., 0.5–4 Hz). The higher components are beyond the heart rate frequency range, thus, are considered to represent higher frequency motion artifact dynamics. Thus, typically, a signal reconstruction is based on only the first two components. A representative signal reconstruction based on the first two components is shown in panel (d) of Figure 6.
3. Adaptive Thresholding Peak Detection
  The next step is to apply adaptive threshold peak detection as described in [5] with the following parameters: 300 ms for refractory period, and α = 0.2. The result of the process is shown in the dashed lines in panel (d) of Figure 6.

#### 2.2.6. New Peak Correction for Non-AF Windows

**Figure 8:**
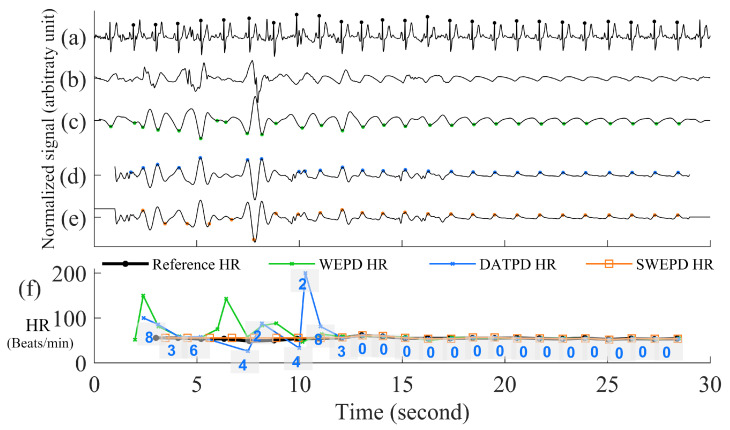
Representative example of the peak correction for NSR. Top panel (**a**) is the reference ECG; panel (**b**) is the raw PPG signal; panel (**c**) is the peak detection output from WEPD; panel (**d**) is the peak detection from DATPD; panel (**e**) is the SWEPD peak correction for NSR with corrected heartbeats (unit: BPM). The bottom panel (**f**) is the heart rate comparison with the reference heart rate, estimated heart rates from WEPD, DATPD, and SWEPD. Region information for each heartbeat of DATPD method is labeled on the heart rate in the bottom panel (**f**).

1. Peak Correction for PAC/PVC Subjects
  Now we are in the step F in Figure 1. The next step involves PAC/PVC detection so that their heartbeats are correctly accounted for. In the event of a mis-detected PAC/PVC beat, the next subsequent beat (let *n* = 1) will be located in the 4th region of the Poincaré plot of the heart rate difference. This is because for a fast PAC/PVC heartbeat the difference of the heart rate at *n* = 1 to the prior normal sinus heart rate at *n* = 0 will be a relatively large but negative value. In addition, the difference between the heart rate at *n* = 1 to that at the next normal sinus beat (*n* = 2) will be a large but positive value. Thus, the beat at *n* = 1 will be located in region 4. This is shown in panel (f) of Figure 7 where the location of the region is provided for each PPG beat. Note in this example that all beats immediately after PAC events are located in the 4th region. When the 4th region along with a HR < 60 BPM are detected, this indicates a missed PAC/PVC beat. We then rectify this problem by using the same approach as detailed in the section Peak Correction for Fast HR AF Segments. Specifically, we used the bandpass [0.5, 10] Hz filtered PPG data to look for the first minimum point in the time location where PAC/PVC occurred. This correction is shown in panel (f) of Figure 7 by the light gray line with “x” marks. With these correction procedures, the PPG-derived heart rates match well with the ECG-derived heart rate, as shown in panel (f) of Figure 7.
2. Heart Rate Correction for NSR Subjects
  For those segments that are detected as NSR after PAC/PVC detection procedures, as shown in step G in the flowchart of Figure 1, we examine if there exist highly deviated heartbeats when they are compared to either the previous or future heartbeats. An example of this is provided in panel (e) of Figure 8. In the Poincaré plot, NSR heartbeats are mostly confined within the zeroth region as there is not much difference in heartbeats from one beat to the next. If there is any heartbeat outside the zeroth region, it is most likely due to a brief episode of motion artifact or bad signal-to-noise ratio, thus, these contaminated or erroneous heartbeats need to be corrected. To correct for the erroneous heartbeats, we take the average heart rate from two neighboring heartbeats of the mis-detected beats, that are located in the zeroth region. If there is no good heartbeat either before or after the noisy heartbeats, we use the mean heart rate of all beats that reside in the zeroth region. In the case where there is a large portion of incorrect beats (e.g., in Figure 8, starting from 2 s to 12 s), we calculate how many beats need to be inserted in the affected time segment. For example, the number of beats that need to be inserted is equal to mean heart rate (computed as beats/s) multiplied by the affected time segment.

#### 2.2.7. Evaluation Methods

Reference ECG heart rates for each 30 s window are automatically calculated by the Biosig toolbox [39], and then they are manually corrected for erroneous beats. The erroneous beats for each 30-s segment were visually inspected and manually corrected, if found. If a R-wave was corrupted from the selected channel of the Holter data, we looked for the same time stamp of the data from the other two channels of Holter, and if the data were clean from either of the two channels, that R-wave information was used instead. If the data from the other two channels were also noisy, we discarded this segment since we needed the entire clean 30-s segment from Holter ECG as it was used as the reference to which PPG heart rate estimates were compared. For one-lead ECG patch data which were used as testing data, we discarded the entire 30-s segment when even a single R-wave was not discernible. Once ECG R-peaks have been detected, the ECG data are down-sampled from 128 Hz to 50 Hz to match the PPG sampling rate. We compared the proposed approach (SWEPD) to WEPD and the DATPD method explained in the Methods section. Four metrics were used to quantify the performance of the peak detection algorithms. The metrics were: (1) averaged value of beat-by-beat root mean squared error (RMSE) between the PPG’s and the reference ECG’s heart rates [7,40], (2) averaged RMSE of the mean heart rates between the PPG and the reference ECG [7,41], (3) false positive (FP) beats [1,4,7,42,43,44], and (4) false negative (FN) beats [1,4,7,42,43,44]. Since peak detection is a single-class classification problem, there are no true negative (TN) values to evaluate. The total number of positive target classes is the number of reference ECG beats in Table 1. We only show the number of extra beats or false positives (FP), and the number of undetected beats or false negatives (FN), in Table 2 and Table 3. Since the heart rate has more clinical significance than does the number of beats, and the evaluation of heart rates also take into account for the incorrect heart rates caused by FP and FN beats, the sensitivity and specificity evaluation of beats are not provided but RMSEs of the heart rate estimations are provided instead.

For calculation of RMSE between the PPG and ECG heart rates for both (1) and (2), a detected PPG peak after an ECG’s R-peak was used. For RMSE calculation, we followed the same approach as the methods proposed by Harju et al. [7] and Parak et al. [45]. For example, if there was only one ECG peak within a PPG beat interval ([t−0.5l, t+0.5l], where *t* is the time when a PPG beat was detected, and *l* is the length of the corresponding interbeat interval (IBI)), we considered this to be the case with no FP or FN peak detection. If there was no ECG peak within a PPG beat interval, this case was labelled as an FP beat. When there was no reference ECG beat, we interpolated the ECG heart rate to 50 Hz, and the time location at which the PPG beat occurred was used as the ECG R-peak, and calculation of RMSE was between PPG heart rate and interpolated ECG heart rate [46]. If there were more than two ECG R-peaks identified within a PPG beat interval, this case was labelled as an FN beat, and the reference heart rate that was closest to the PPG heart rate was used to calculate the RMSE. We summed all squared error values for each 30-s segment and calculated the averaged RMSE for a given rhythm type (e.g., NSR, normal heart rate AF, fast heart rate AF, and PAC/PVC). The calculation of average beat-to-beat RMSE and RMSE of the mean IBI was performed again on all the segments. For the RMSE of the mean heart rate, we calculated the squared error of the mean of PPG heart rate and the mean of reference ECG heart rate for each 30 s window, and then calculate averaged RMSE for all 30 s windows for a given arrhythmia type. We also calculated the average beat-to-beat RMSE and RMSE of the mean IBI for all segments. The total number of FP beats and FN beats is the sum of all FP and FN beats from each 30 s window.

**Table 2:** Evaluation of Proposed Peak Detection Methods on Training Dataset (Samsung Simband).

Evaluation Method	Subject Type	SWEPD ^1^	WEPD ^2^	DATPD ^3^	Method 1 ^4^	Method 2 ^5^	Method 3 ^6^	Method 4 ^7^	Method 5 ^8^	Method 6 ^9^	Method 7 ^10^
Average beat-to-beat RMSE (BPM/segment) (IBI in millisecond)	NSR	2.21 (29.64)	5.65 * (61.98 *)	8.77 * (88.62 *)	12.10 * (264.60 *)	11.55 * (226.54 *)	12.23 * (118.68 *)	12.70 * (102.66 *)	7.61 * (124.89 *)	12.54 * (113.30 *)	8.32 * (79.54 *)
	PAC/PVC	12.53 (97.85)	10.48 * (135.28 *)	21.02 * (300.94 *)	27.34 * (526.13 *)	28.30 * (530.20 *)	15.19 (266.84 *)	14.20 (269.08 *)	16.34 (337.61 *)	16.65 (285.17 *)	15.48 (269.24)
	Basal AF	7.69 (101.58)	7.69 (101.58)	22.11 * (276.09 *)	22.67 * (397.77 *)	23.91 * (426.28 *)	22.55 * (208.66 *)	23.97 * (214.24 *)	14.31 * (245.26 *)	24.04 * (217.47 *)	12.34 (168.78)
	AF with RVR	35.49 (164.28)	34.31 * (289.99 *)	50.52 * (480.23 *)	53.95 * (547.95 *)	61.52 * (713.14 *)	40.25 * (295.21 *)	41.18 * (296.12 *)	47.27 * (440.27 *)	43.96 * (335.49 *)	43.33 * (327.31 *)
RMSE of the mean HR (BPM/segment) (IBI in millisecond)	NSR	0.46 (5.78)	2.27 * (23.62 *)	4.32 * (33.15 *)	10.77 * (243.04 *)	12.00 * (260.22 *)	29.53 * (139.97 *)	27.28 * (139.91 *)	3.92 * (42.26 *)	23.00 * (133.40 *)	9.92 * (67.37 *)
	PAC/PVC	3.06 (23.30)	8.63 * (94.90 *)	10.33 * (142.96 *)	20.17 * (332.85 *)	26.31 * (400.47 *)	7.77 * (91.67 *)	7.77 * (90.33 *)	10.37 * (205.62)	8.43 * (109.92 *)	8.51 * (92.24 *)
	Basal AF	3.81 (44.18)	3.81 (44.18)	10.95 (97.57 *)	11.25 * (197.11 *)	14.81 * (242.83 *)	40.03 * (178.34 *)	33.83 * (179.33 *)	5.82 (86.07)	34.42 * (187.13 *)	9.90 (73.45)
	AF with RVR	16.90 (87.20)	31.38 * (231.25 *)	41.71 * (381.64 *)	42.75 * (397.93 *)	51.63 * (532.93 *)	21.11 (162.94 *)	21.63 (163.91 *)	33.30 * (269.30 *)	25.09 * (193.96 *)	26.77 * (197.89 *)
Extra Beats (Beats)	NSR	50	85 *	116 *	205 *	63 *	677 *	507 *	172 *	529 *	211 *
	PAC/PVC	4	7	22	7	3	24 *	16	4	24 *	13
	Basal AF	9	9	55 *	20	7	136 *	134 *	10	162 *	30 *
	AF with RVR	114	12 *	14 *	6 *	1 *	35 *	44 *	8 *	23 *	22 *
Undetected Beats (Beats)	NSR	18	30 *	68 *	697 *	637 *	195 *	54 *	168 *	70 *	61 *
	PAC/PVC	19	63 *	126 *	222 *	267 *	65 *	63 *	92 *	74 *	64 *
	Basal AF	17	17	94 *	128 *	166 *	11	15	50 *	17	19
	AF with RVR	33	367 *	442 *	523 *	617 *	297 *	310 *	420 *	342 *	341 *

^1^ SWEPD: Sequence with waveform envelope peak detection; ^2^ WEPD: Waveform envelope peak detection; ^3^ DATPD: Differentiator-adaptive threshold peak detection (Lázaro et al. [5]); ^4^ Method 1: ‘Peak’ detection from Shin et al. [4]; ^5^ Method 2: ‘Foot’ detection from Shin et al. [4]; ^6^ Method 3: Local maxima detection in [47] and Elgendi’s paper [1]; ^7^ Method 4: Local minima detection from method 1 in [47] and Elgendi’s paper [1]; ^8^ Method 5: first derivative and adaptive thresholding method in Li et al. [42] and Elgendi’s paper [1]; ^9^ Method 6: updated slope sum function with adaptive thresholding method in Zong et al. [43] and Elgendi’s paper [1]; ^10^ Method 7: event-related moving averages with dynamic threshold method in Elgendi et al.’s paper [1]; * *p*-value < 0.05 between SWEPD and the comparison method.

**Table 3:** Evaluation of Proposed Peak Detection Methods on Independent Testing Dataset (Samsung Gear S3 and Samsung Galaxy Watch 3).

Evaluation Method	Subject Type	SWEPD ^1^	WEPD ^2^	DATPD ^3^	Method 1 ^4^	Method 2 ^5^	Method 3 ^6^	Method 4 ^7^	Method 5 ^8^	Method 6 ^9^	Method 7 ^10^
Average beat-to-beat RMSE (BPM/segment) (IBI in millisecond)	NSR	3.69 (41.16)	5.56 (64.13 *)	6.56 (94.08 *)	10.72 * (242.75 *)	10.94 * (240.94 *)	5.96 (66.73 *)	7 * (82.2 *)	21.18 * (1027.26 *)	44.3 * (605.89 *)	7.07 * (78.37 *)
	PAC/PVC	8.2 (95.21)	8.2 (106.29)	17.25 (241.06)	17.36 * (341.21 *)	15.64 * (303.75 *)	8.35 (105.38)	10.11 (122.93)	27.61 * (1869.61 *)	45.11 * (702.63 *)	9.04 (107.01)
	Basal AF	12.38 (98.17)	16.31 (148.87 *)	29.4 * (303.03 *)	40.63 * (571.8 *)	32.83 * (503.88 *)	14.74 (125.94)	15.56 (134.85)	30.27 * (665.65 *)	49.31 * (960.74 *)	14.06 (123.15)
	AF with RVR	23.4 (152.5)	28.45 * (212.72 *)	41.48 * (368.73 *)	53.86 * (640.06 *)	45.43 * (502.85 *)	26.71 (189.85 *)	25.37 (180.24 *)	42.11 * (501.88 *)	51.25 * (793.82 *)	23.06 (161.05)
RMSE of the mean HR (BPM/segment) (IBI in millisecond)	NSR	4.12 (25.54)	6 * (62.2 *)	5.22 * (53.63 *)	12.58 * (335.74 *)	12.24 * (295.5 *)	7.45 * (67.99 *)	8.56 * (70.39 *)	10.43 * (788.84 *)	58.6 * (629.45 *)	8.58 * (67.08 *)
	PAC/PVC	7.1 (115.75)	5.08 * (105.46 *)	6.95 * (130.79 *)	14 * (302.67 *)	12.02 * (286.72 *)	8.02 (110.21)	9.03 * (113.28 *)	15.4 * (1432.1 *)	52.56 * (693.07 *)	7.12 (104.77)
	Basal AF	7.19 (50.76)	7.79 * (78.36 *)	13.82 * (143.3 *)	30.24 * (448.42 *)	22.4 * (515.48 *)	8.12 * (64.65 *)	7.52 * (65.07 *)	16.69 * (462.89 *)	35.16 * (836.51 *)	8.15 (95.21)
	AF with RVR	9.48 (58.79)	12.06 * (92.88 *)	27.88 * (241.39 *)	43.81 * (499.38 *)	33.84 * (356.92 *)	13.23 (95.11 *)	12.06 (93.82)	27.47 * (297.5 *)	23.15 * (316.2 *)	8.06 (70.83)
Extra Beats (Beats)	NSR	27	234 *	163 *	110 *	155 *	345 *	390 *	947 *	9620 *	477 *
	PAC/PVC	192	93 *	43 *	27 *	64 *	109 *	136	177	2936 *	150
	Basal AF	604	427	2492 *	120 *	254 *	465 *	786 *	363 *	5478 *	842 *
	AF with RVR	43	33	99 *	1 *	25	32	94 *	15 *	432 *	105 *
Undetected Beats (Beats)	NSR	132	387 *	498 *	2306 *	2118 *	402 *	478 *	5361 *	3006 *	444 *
	PAC/PVC	197	315 *	506 *	1290 *	1057 *	314 *	370 *	3074 *	1572 *	320 *
	Basal AF	978	1763 *	6467 *	10103 *	6802 *	1482 *	1692 *	6512 *	9296 *	1521 *
	AF with RVR	236	354 *	864 *	1213 *	929 *	322	371 *	820 *	946 *	321 *

^1^ SWEPD: Sequence with waveform envelope peak detection; ^2^ WEPD: Waveform envelope peak detection; ^3^ DATPD: Differentiator-adaptive threshold peak detection (Lázaro et al. [5]); ^4^ Method 1: ‘Peak’ detection from Shin et al. [4]; ^5^ Method 2: ‘Foot’ detection from Shin et al. [4]; ^6^ Method 3: Local maxima detection in [47] and Elgendi’s paper [1]; ^7^ Method 4: Local minima detection from method 1 in [47] and Elgendi’s paper [1]; ^8^ Method 5: first derivative and adaptive thresholding method in Li et al. [42] and Elgendi’s paper [1]; ^9^ Method 6: updated slope sum function with adaptive thresholding method in Zong et al. [43] and Elgendi’s paper [1]; ^10^ Method 7: event-related moving averages with dynamic threshold method in Elgendi et al.’s paper [1]; * *p*-value < 0.05 between SWEPD and the comparison method.

## 3. Results

Among 35 participants’ data, we identified 271 30-s segments as analyzable data. The adjudication of the type of arrhythmias was performed by the UMass group and their decisions were based on the reference ECG data. The adjudication of the types of arrhythmias are shown in Table 4. Among 35 participants, 23 participants were identified as NSR, and there were 5 participants with PAC/PVC. Among 5 subjects with PAC/PVC, 2 subjects had both NSR segments and PAC/PVC segments. Among 9 subjects with AF, there were 5 participants with basal heart rate AF, and 4 participants with RVR AF. Among the total of 271 30-s segments, we identified 190 segments as NSR with a corresponding 5740 ECG beats, 28 segments as PAC/PVC with a corresponding 897 ECG beats, 25 segments as basal heart rate AF with a corresponding 818 ECG beats, and 28 segments as fast heart rate AF with a corresponding 1334 ECG beats. The average PPG beats per 30-s segment were: 30.21 beats for NSR, 32.04 beats PAC/PVC, 32.72 beats for the basal heart rate AF, and 47.64 beats for the fast heart rate AF.

### 3.1. Results of AF with RVR Detection

The classification into basal and fast heart rates by both the adjudicators and the SWEPD method is shown in the confusion matrix of Table 5. These results were derived from the scatter plot shown in Figure 5. Selecting the mean distance feature’s threshold value as noted by the demarcation line in Figure 5, we obtain good separation between the basal and fast AF heart rates. Based on the chosen threshold value of the scatter plot, only 2 of the 28 RVR AF heart rates were misclassified as normal/basal heart rates.

### 3.2. Results of Proposed Peak Detection Method

Table 2 shows a comparison of the proposed comprehensive method (SWEPD) to the WEPD and DATPD methods for all arrhythmia types examined in this work. In addition, comparison of the proposed SWEPD to seven other methods that were designed for NSR is shown in Table 2. The methods 1 and 2 are the ‘peak’ and ‘foot’ detection, respectively, as described in Shin et al. [4]; method 3 and method 4 are detection of the local maxima and local minima, respectively, as described in [47] and Elgendi et al. paper [1]; method 5 is based on the first derivative and adaptive thresholding method in Li et al. [42] and Elgendi et al. [1]; method 6 is based on the updated C code implementation of the slope sum function with an adaptive thresholding method by Zong et al. [43]; method 7 uses the event-related moving averages with a dynamic threshold method as described in Elgendi et al. [1]. The implementation of the seven comparison methods is available on https://biosignal.uconn.edu/resources/(accessed on 30 June 2021).

In this table, we show both RMSE values of heart rates and IBI for all arrhythmias considered for our three methods and the seven other compared methods. The comparison serves two purposes. First, the WEPD method—a new peak detection algorithm also used for a portion of the SWEPD method—is compared to the DATPD method and seven other methods. The DATPD method is one of the accurate PPG peak detection algorithms, previously developed by Lázaro et al. [5]. Second, our proposed SWEPD approach is also compared to both the WEPD, DATPD methods, and seven other methods.

The beat-to-beat average RMSE values of the SWEPD method are all lower than the WEPD, DATPD and seven other methods for the arrhythmia types listed in Table 2. The two biggest improvements in the beat-to-beat average RMSE are seen for the NSR and basal heart rate AF detection (75% and 65% reduction in the RMSE over the DATPD method, respectively), followed by the PAC/PVC and RVR heart rate AF detection.

Moreover, while the number of extra beats detected by the SWEPD method is higher than for the WEPD, DATPD and seven comparison methods for the RVR heart rate AF, the number of missing beats detected is significantly lower for all rhythm types.

We also computed RMSE of the mean HR as shown in the second row of Table 2. The proposed SWEPD approach provides the lowest RMSE of the mean HR values for all rhythm types, followed by the WEPD, method 5, and DATPD methods. The decrease in the RMSE of the mean HR values is as high as 60% for the RVR heart rate AF when compared to the DATPD method. The reduction in the RMSE of the mean HR is even more staggering for PAC/PVC beats, as we observe 70% decrease with the proposed SWEPD detection approach over the DATPD method.

The RMSE values of IBI, for all types of arrhythmias considered in this work, showed significantly lower values for SWEPD when compared to both WEPD and DATPD. The IBI values are shown in parentheses in Table 2. Similar to RMSE values of beat-to-beat and mean HR, the corresponding IBI values are nearly 7 times lower with the SWEPD when compared to DATPD for all arrhythmias considered.

When compared with DATPD method, the method 5 had better results for NSR subjects and the method 7 provided better results when compared to most of the evaluation method albeit if produced significant number of extra beats of NSR subjects, our SWEPD and WEPD methods were far superior when compared to the seven methods as they are mainly designed to handle NSR and not AF with RVR or fast HR PAC/PVC, while the performance metrics of DATPD algorithm are not as good as those of WEPD, we use it for non-AF segments as it is not able to detect PAC/PVC beats. As shown in Figure 1, the next step after DATPD is to detect for the presence of PAC/PVC, and if the PAC/PVC-associated patterns exist, they can be used to detect extra beats associated with PAC/PVC.

### 3.3. Results of Proposed Peak Detection Method on Testing Dataset

Among 25 participants’ data, 2112 clean 30-s segments were randomly selected for independent testing, which is nearly eight times the number of segments used for the training dataset. The adjudication results of the types of arrhythmias are shown in Table 1. Details of the arrhythmia distribution for each subject can be found in Table A1 in the Appendix A section. Among 25 participants, there were 8 non-AF subjects and 17 AF subjects. Even though we have fewer NSR subjects compared to the training dataset, we have almost twice as many AF subjects for the testing data. Among 8 non-AF subjects, 7 subjects had both NSR and PAC/PVC segments and 1 subject had only NSR segments. Among the 17 AF subjects, 4 subjects had both basal AF and RVR AF segments, 12 subjects had only the basal AF, and 1 subject had RVR AF. One of the 25 subjects used a Galaxy Watch 3 while the other 24 subjects used the Gear S3. In total, there were 824 segements of NSR, 352 segments of PAC/PVC, 869 segments of basal AF, and 67 segments of AF with RVR. The number of AF segments is comparable with the number of NSR segments in the testing dataset, while in the training dataset, the number of AF segments is only 13% of the NSR segments. The PAC/PVC segments in the testing dataset are nearly 1.5 times higher than in the training dataset. The average number of reference ECG beats per segment are 27.42, 25.60, 34.66, and 43.42 BPM for NSR, PAC/PVC, basal AF and AF with RVR, respectively.

**Figure 9:**
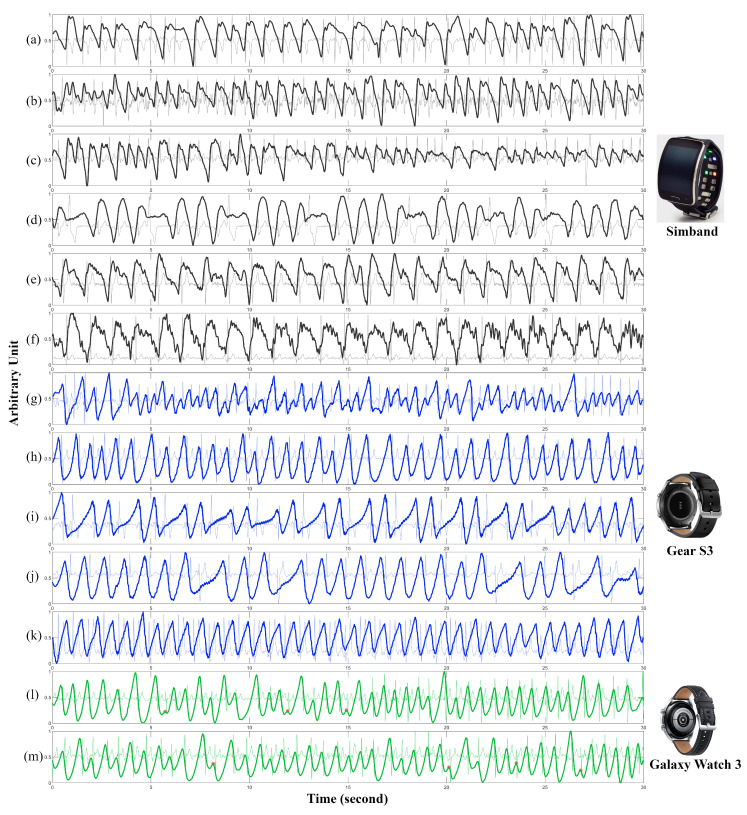
Example signal traces from three different types of watches and different types of arrhythmias. PPG signals are shown in the foreground, and reference ECG signals are plotted in the background. Panels (**a**–**f**): raw PPG signals collected with Simband and from AF (**a**–**c**), PVC (**d**), NSR (**e**,**f**). Panels (**g**–**k**): raw PPG signals (baseline wander removed) collected with Gear S3 (2016) and from AF (**g**,**h**), PAC (**i**), PVC (**j**), and NSR (**k**). Panels (**l**,**m**): raw PPG signal (baseline wander removed) collected with Galaxy Watch 3 (2020) and from AF participants. The dicrotic-notch like PPG peaks marked in red in panels (**l**,**m**) should be detected as true peaks as they correspond to actual AF beats shown in the background reference ECG.

We show the results of independent testing using our proposed PPG peak-detection algorithms along with seven other published algorithms in Table 3. We want to stress that no further tuning of the algorithm was done with the testing dataset for PPG peak detection. The training of the algorithms was solely based on Simband smartwatch data. Moreover, the testing dataset consists of PPG data from two different models of Samsung smartwatches: Gear S3 and Galaxy Watch 3. The difference in the morphological waveforms from these three different models of smartwatch can be clearly seen in Figure 9. The panels with black lines in Figure 9 show that PPG recorded with Simband capture finer details such as the dicrotic notch but it also contains high frequency noise, while the panels with blue and green lines in the same figure show that the PPG recorded on Gear S3 and Galaxy Watch 3 are heavily filtered since they exhibit smoother waveforms. Given these characteristics of the Gear S3 and Galaxy Watch 3, we changed the cutoff frequency of the bandpass filter from [0.5, 5] Hz to [0.5, 8] Hz which is the same cutoff frequency used in Elgendi’s method; we did not use a moving average filter to avoid further loss of details in the PPG waveforms.

Table 3 shows the independent testing results. SWEPD has the overall best performance as it provided the lowest RMSE values for most cases involving various arrhythmias. For the average beat-to-beat RMSE (beats/min) in the first row of Table 3, SWEPD had 47.8%, 9.3%, and 13.6% lower RMSE values than did the next best method (method 7) on NSR, PAC/PVC, and basal AF, respectively. Only for AF with RVR, SWEPD had 1.5% greater RMSE value than did method 7. In the second row of Table 3, which shows RMSE of the mean heart rates as well as the mean IBI values, SWEPD again provided lower than method 7’s RMSE values for NSR, PAC/PVC, and basal AF, but for AF with RVR, SWEPD had 15% higher RMSE than did method 7. For PAC/PVC rhythm, the best performer was WEPD for the RMSE of the mean heart rate estimation followed by SWEPD and method 7. Method 7 is sensitive to peaks on the upper portion of the PPG waveform, thus, it detects more beats as heart beats on AF with RVR subjects. However, the consequence of detecting additional beats is that it also detects many extra false positive beats for other rhythms such as NSR, PAC/PVC, and basal AF. SWEPD had the lowest number of undetected beats among all four types of arrhythmias while not having too many extra beats detected when compared to method 7. Method 1 had the lowest extra beats detected for all rhythms other than NSR followed by SWEPD. However, SWEPD was the best performer for having the lowest number of undetected beats.

## 4. Discussion

In this work, we illustrate a sequence of algorithms to discriminate various rhythms from PPG data, including NSR, PAC/PVC, and basal and RVR heart rate AF, so that more accurate PPG peak estimation can be attained. To clarify, the compared methods were implemented using the parameter values reported in the literature. However, it is possible that the performance metrics may improve if the parameters are optimized for the signals used in this paper. We have shown that our SWEPD approach provides a reduction in error over 63% that of an existing peak detection method for the rhythms noted above. To date, we are not aware of an algorithm that is able to automatically calculate appropriate HR when it varies due to different types of arrhythmias. When a peak detection algorithm that is primarily designed for NSR is applied to rhythms other than NSR, significant estimation errors in heart rates occur. One algorithm incorporated into our new combined algorithmic approach illustrated in Figure 1 (SWEPD) is a new peak detection algorithm (WEPD). Our algorithm also incorporates a new peak correction method to compensate for the fast HR that can occur in AF with RVR, which is a type of AF that has not been previously reported using smartwatches. Moreover, because this rhythm results in large and sudden changes from low HR to high HR, a PPG peak detection method specifically designed for NSR would have difficulty in capturing these large variations in HR. As other PPG peak detection algorithms are mainly designed to handle NSR and AF with normal heart rates, they would lead to large heart rate or interbeat interval estimation errors with PAC/PVC and AF with RVR. This is clearly shown in Table 2, as DATPD, which was primarily designed for NSR, degrades significantly for PAC/PVC and AF with RVR. We showed that SWEPD is able to improve the accuracy of heart rate estimation, largely due to significant reductions in false negative peaks detected during arrhythmia.

Harju et al.’s method [7], method 5 [42], and method 7 [1] provide good peak detection accuracy for both NSR and AF, however, it is less accurate when there is PAC/PVC or AF with RVR [7]. It is our opinion that DATPD, originally proposed by Lázaro et al. [5], and Harju’s peak detection method [7], method 5 [42], and method 7 [1] are similar in their implementation and results. As seen in Table 2, the IBI with our SWEPD has approximately 3 times less beat-to-beat RMSE than does DATPD for all rhythms (NSR, AF, PAC/PVC, and AF with rapid ventricular response). Our SWEPD has nearly 7 times lower mean RMSE of IBI than does DATPD for PAC/PVC rhythms. Note that Harju’s method [7], method 5 [42], and method 7 [1] are not designed to handle PAC/PVC and AF with rapid ventricular response so they will also have large IBI RMSE values. Note that most other approaches involve either removing PAC/PVC beats entirely or interpolating between two non-PAC/PVC beats. However, when there are many PAC/PVC beats, these approaches can lead to inaccurate HR results.

### 4.1. AF with RVR Detection Accuracy

Certainly, significant heart rate estimation error occurs when a peak detection algorithm that is designed for basal heart rate NSR is applied to AF with RVR. The evidence is seen in Table 2, as both the WEPD and DATPD peak detection algorithms show greater estimation error than does the proposed SWEPD approach. The key advancement for detecting RVR versus basal heart rates is a novel use of a Poincaré plot to extract features that can discriminate between the two rhythms. Figure 5 illustrates good separation between the basal and RVR heart rates using the features noted in the plot. Note also the significant reduction in the false negative peak detection with our approach when compared to either the WEPD or DATPD peak detection methods. The number of missing peaks was 442, 367, and 33 for the DATPD, WEPD, and the proposed SWEPD approach, respectively. Consequently, as expected, the reductions in the RMSE of the mean HR for both AF with RVR and PVC/PAC were more than 60% for these two arrhythmias via the proposed SWEPD approach when compared to either the DATPD or WEPD methods.

We had few cases of AF with RVR segments in this work. Hence, there were not enough training and testing datasets for AF with RVR. However, when more data become available, the algorithm’s performance can be further improved.

The clinical relevancy of detecting AF with RVR is that with accurate hear rate information, clinicians can prescribe appropriate drugs to reduce fast beating heart rates which can be detrimental if prolonged [32]. For example, the fast ventricular rate during AF reduces ventricular filling and stroke volume, which can lead to heart failure and can cause a tachycardia-induced cardiomyopathy [32].

### 4.2. Peak Detection Accuracy for the WEPD and SWEPD Approaches

As shown Table 2, WEPD provides lower RMSE when compared to DATPD for all arrhythmias. Moreover, the number of undetected peaks detected with WEPD is considerably lower when compared to DATPD. The number of false positive detected peaks between the two methods are comparable, however. Consequently, the RMSE is lower for WEPD than for DATPD for all arrhythmias considered in this work. The key feature of WEPD which led to better performance than DATPD is the use of an adaptive envelope approach to better discriminate fast heart rates associated with AF and PAC/PVC rhythms.

When WEPD is combined with the capability to discriminate between RVR and basal heart rates (the SWEPD approach), we observe even more dramatic improvements in the RMSE and a significant reduction in the number of false negative detected peaks for all arrhythmias, as shown in Table 2, when our new approach is compared against either WEPD or DATPD. The reduction in the RMSE of the mean heart rates of the fast heart rate AF and PAC/PVC rhythms is more than 60% with the proposed SWEPD approach when compared to either WEPD or DATPD. The reduction in the number of false negative peaks detected is even greater with the SWEPD approach when compared to either WEPD or DATPD. These improvements are largely due to SWEPD’s compensation for the fast heart rates that are often associated with PAC/PVC and AF. As previously stated, the fast heart rates were discriminated from basal heart rates using features derived from a novel use of Poincaré plots.

Clinically, accurate HR estimation is of critical importance for AF subjects since HR control is a key treatment outcome. As seen in Table 2, neither WEPD nor DATPD were able to provide accurate mean HR information for participants with AF who had high ventricular response rates. When an ECG is used to detect AF, a reliable mean heart rate can also be obtained. However, with a PPG signal, accurate mean heart rates are not attainable with prior peak detection algorithms. Given that we are now able to provide more reliable estimates of mean heart rates for participants in AF via the proposed novel approach, we have potentially enabled clinicians to use PPG data from mobile and wearable devices for HR monitoring of patients with AF. SWEPD’s computational time in MATLAB is approximately 0.21 s (Xeon E5-2650, 64 GB RAM 2400 MHz DDR4), and its running time on a Samsung Galaxy Watch S3 is around 5 s.

### 4.3. Limitation of Small Sample Size

As this is a preliminary study to design a real-time peak detection algorithm for the clinical trial, the number of data samples was limited. However, to compensate for a limited data sample size, we have tested the algorithm using independent datasets acquired from Gear S3 and Galaxy Watch 3 models as the training was solely based on Simband. More importantly, the results were based on fixing all parameters derived from the training datasets from Simband and no further tuning was done for the testing datasets from Gear S3/Galaxy Watch 3. Moreover, the testing data were larger than the training data, hence, the proposed algorithm showed good generalizability as it remained accurate for a wide variety of arrhythmia in the testing data. When our clinical trial is finished, we hope to obtain more diverse data which will allow even greater scrutiny and performance evaluation of our algorithm against the seven other compared methods.

Thresholding used may not be the most robust approach as it is based on the available data. However, we do have an ample number of training and testing datasets collected with different smartwatches (Simband, Gear S3, and Galaxy Watch 3). As stated earlier, training of the algorithm was based on only Simband data, whereas the testing datasets were from Gear S3 and Galaxy Watch 3, which have different signal quality than Simband. Hence, we have confidence that the chosen thresholds will remain valid even for additional unseen PPG data from Gear S3 and Galaxy Watch 3. Certainly, machine learning approaches may be a better approach but they are also dependent on the training data.

## 5. Conclusions

In this paper, we introduced two algorithms that are designed to provide better peak detection accuracy for the rhythms NSR, PAC, PVC, and AF. The first algorithm, WEPD, was shown to provide better peak detection accuracy over the compared method (DATPD), which has been shown to provide accurate results for NSR. The second method combines WEPD and a novel approach to discriminate between RVR and basal heart rates. Moreover, by sequentially querying various different scenarios, as described in the flowchart of Figure 1, our proposed SWEPD method is able to automatically determine which algorithms are most applicable for the type of rhythm presented. These key advancements have resulted in both significant reductions in the RMSE values and the number of false negative detected peaks with our proposed SWEPD approach when it is compared to either the WEPD on its own, or DATPD. The IBI error reduction with SWEPD when compared to DATPD was nearly 7 times lower with the fast heartbeats which are often associated with PAC, PVC, and in many cases AF. To our knowledge, this is one of the first studies to report a real-time PPG peak detection algorithm that is shown to be effective across several generations of models of Samsung smartwatches. In addition, the novelty of the algorithm is that it is effective not only for NSR, which is all most other algorithms have been tested on, but also for complicated PPG morphologies including AF and PAC/PVC. The future work will be further validation of our approach as we collect new data with the ongoing clinical trial. Once the clinical trial is over, and once all data have been analyzed, we hope to provide additional results thereafter.

## 6. Patents

A patent application is pending based on the results reported in this manuscript. United States Application No. 17/364,684. Filed date: 30 June 2021. Title: Heart Condition Treatment and Analysis. Inventors: Ki H. Chon, Dong Han, Syed Khairul Bashar, and Fahimeh Mohagheghian. Applicant: University of Connecticut. Attorney Docket No.: 38100.0003U5.

## Figures and Tables

**Table 1:** Independent Testing Dataset Information.

	Subject Type			
	**NSR**	**PAC/PVC**	**Basal AF**	**AF with RVR**
Subjects	8	7	16	5
30-s segments	824	352	869	67
Reference ECG Beats	22,597	9011	30,117	2909

**Table 4:** Training Dataset Information.

	Subject Type			
	**NSR**	**PAC/PVC**	**Basal AF**	**AF with RVR**
Subjects	23	5	5	4
30-s segments	190	28	25	28
Reference ECG Beats	5740	897	818	1334

**Table 5:** Confusion Matrix for Training Stage of AF with RVR Detection.

		Actual Class (30-s Segments)	
		**Basal AF**	**AF with RVR**
Predicted Class	Basal AF	25	2
	AF with RVR	0	26

## Data Availability

The training dataset, Simband dataset is available for download on our lab’s website: https://biosignal.uconn.edu/resources/ (accessed on 30 June 2021). The testing dataset will be available for download after the data organizing of the clinical trial is finished.

## Abbreviations

The following abbreviations are used in this manuscript:

PPG	photoplethysmogram
NSR	normal sinus rhythm
PAC	premature atrial contraction
PVC	premature ventricle contraction
AF	atrial fibrillation
RVR	rapid ventricular response
HR	heart rate
SNR	signal-to-noise ratio
WEPD	waveform envelope peak detection
DATPD	differentiator-adaptive threshold peak detection
SWEPD	the overall sequence using WEPD
UMMC	University of Massachusetts Medical Center
BPM	beats per minute
VFCDM	variable frequency complex demodulation method
RMSE	root mean squared error
IBI	interbeat interval

## Appendix A

**Table A1:** Detailed Information of Independent Testing Dataset.

Index	UID	NSR	PAC/PVC	Basal AF	AF with RVR	Watch Model
1	002	102	7	0	0	Gear S3
2	005	107	194	0	0	Gear S3
3	017	0	0	167	3	Gear S3
4	029	31	3	0	0	Gear S3
5	038	241	117	0	0	Gear S3
6	045	232	4	0	0	Gear S3
7	054	98	25	0	0	Gear S3
8	301	0	0	17	0	Gear S3
9	302	0	0	2	0	Gear S3
10	305	0	0	10	0	Gear S3
11	306	0	0	1	1	Gear S3
12	307	0	0	39	0	Gear S3
13	310	0	0	6	0	Gear S3
14	311	0	0	19	0	Gear S3
15	312	0	0	3	0	Gear S3
16	318	0	0	0	8	Gear S3
17	319	0	0	13	0	Gear S3
18	320	0	0	8	0	Gear S3
19	321	0	0	11	0	Gear S3
20	322	0	0	7	0	Gear S3
21	324	0	0	6	0	Gear S3
22	325	13	0	0	0	Gear S3
23	327	1	6	0	0	Gear S3
24	329	0	0	10	5	Gear S3
25	400	0	0	550	50	Galaxy Watch 3
	Total	824	352	869	67	
